# Scandinavian Emergency Medicine – A toddler steadily walking but still not running

**DOI:** 10.1186/1757-7241-16-6

**Published:** 2008-08-19

**Authors:** Maaret Castrén

**Affiliations:** 1 Professor of Emergency Medicine, Department of Clinical Science and Education, SÖS, Karolinska Institutet, Stockholm, Sweden

## 

I recently read an editorial that reflected upon the past 25 years of emergency medicine [1]. The mantra of the emergency medicine field is "you have to know a lot more than a little about everything". Emergency medicine is extremely fascinating, but may also be very frightening to newly graduated colleagues who are just beginning their careers. Scandinavian universities are still not teaching emergency medicine as a discipline; however, this obstacle does not prevent us from publishing a high quality journal from which young doctors can read.

Another editorial [2] focuses on the development of intensive care medicine during the past 25 years, stating "Intensive care has established its identity and is an acknowledged speciality in medicine". This established identity is the current goal for the much younger discipline of emergency medicine, of which it has been said: "It has been small steps in the right direction. Some mistakes but mostly good things" [2]. A solid, academic, evidence-based foundation in emergency medicine will provide the field with critical protocol and decision-making knowledge. We have a good start for an academic platform in Scandinavia, and The Scandinavian Journal of Trauma, Resuscitation, and Emergency Medicine (SJTREM) is an important resource in supporting this development.

There are only two professors of emergency medicine in Norway and one in Sweden, and emergency medicine is defined as a specific speciality only in Sweden. While Södersjukhuset in Stockholm, the home of the Swedish professor, has had emergency physicians since the late 1990s, emergency medicine officially did not become a subspecialty in Sweden until 2006. There are strong movements in both Finland and Denmark toward the same development, and Norway has begun discussing the need for a defined emergency medicine field. In this regard, we in the Scandinavian countries support each other as best we can. The fact that the president of the European Society of Emergency Medicine is from Sweden will be helpful as we try to reach the goal of emergency medicine as a speciality. A Scandinavian journal is an excellent forum for these discussions.

When I began my career approximately 30 years ago, there was no pulseoximetry, CPAP, CT, MRI, or even US, and no thrombolysis or PCI, laparoscopic surgery, GPS or mobile phones. I wonder what they will write about in the next 25 years, and I dream about the device that they use in the TV show Star Trek. Using this device, doctors would simply scan the patient to receive prompt vital signs, blood results, and diagnoses. Simple and easy, no brain work or knowledge necessary. But wait, stop! Brain work is the most fun part. The fact that you have been able to solve a mystery, such as an unknown diagnosis, and discovered a conclusion or at least a treatment that makes the patient feel better is the best part of the whole profession. Reading about the work of others inspires you to work harder and provides new ideas about how to work more efficiently. A journal is a good way to inform the world of your work and thoughts, and we need to share this information to become a stronger field of medicine.

We have a young but very strong emergency medicine culture in Scandinavia. In the field of resuscitation, our tiny countries have performed solid research. Together, we have addressed post-resuscitation care as an important, and now well-established, link in the Chain of Survival. The majority of research regarding dispatching protocols comes from our countries. An exiting study that randomised patients to an experimental group receiving adrenalin or another group that did not even receive an i.v. during the resuscitation protocol was recently completed, and we are all eager to learn of the results. Anyone who works in the field of pre-hospital care has knowledge of the Utstein formulas. This little monastery name in Stavanger serves as a symbol of quality work, and many papers drafted from Utstein meetings have provided us with a framework to understand results from studies all over the world. We also have unique patient registries that allow us to follow our patients from birth until death, providing years and years of potential data, which is a goldmine for epidemiological studies.

What will be written about the SJTREM in 25 years? Hopefully, it will resemble what Sternbach wrote on the recent 25^th ^anniversary of The Journal of Emergency Medicine, which is that most of the articles in the first issue are relevant to the practitioner today meaning year 2033 for this youngster. By working together as a discipline, I hope that we can make this wish come true and, at the same time, make emergency medicine an established part of everyday medicine in Scandinavia as well as all over the world.

## Competing interests

The author declares that they have no competing interests.
